# Therapeutic Notes

**Published:** 1897-10-16

**Authors:** 


### Therapeutic Notes.

### PYOKTANIN.

One of the most interesting of the recent synthetic remedies which we possess is blue pyoktanin or methylene (not methyl) blue. It was first employed by Mosetig for cancer, both of epithelial and other forms; but in the last two or three years it has been utilised for various diseases. Thus, in a very recent number of The Hospital, we noticed its employment for the diagnosis of latent kidney lesions. From the rapidity of its elimination has been found to be a measure of the power of the kidneys and of their activity in getting rid of noxious matters. A peculiarity of the drug is seen in its turning the urine where the kidneys are healthy to a deep blue within five hours. Netchaieff and others have found that it can be used beneficially in cystitis, gonorrhœa, and Bright's disease; and instances of the disappearance of albumen and casts when it has been employed are recorded. Moore[1] claims that it has cut short the acute stage of gonorrhœa and lessened the pain. The dose is from one to five grains daily, divided into pills or as a solution, but care must be taken lest irritation of the bladder be caused. In small doses it seems to exercise a valuable antiseptic effect in curing chronic cystitis. But its value is not confined to urinary affections. Berthier finds it a powerful remedy in gastric hyperacidity with pain and vomiting. Both here and in many other cases it is desirable to stop the drug after several days, and to give a short rest before resuming it. Finally, it has been employed with some success in malaria where quinine was undesirable; and for the relief of nervous pains, such as certain forms of migraine and neuralgia, and in a few instances it has effected a permanent cure as well as temporary relief of these pains.[2]

[1] B. Med. Journal, Jan. 16. [2] Med. Record, Ap. 24.

### ORPHOL.

Beta-naphtholbismuth, or orphol, is reported as a valuable agent in the diarrhœa of tuberculous patients. Ordinary astringents and opiates have little effect in lessening the colicky pains with liquid blood-streaked stools which accompany intestinal ulceration of this kind. We need both an antiseptic and an astringent which will render the mucous membrane a bad culture ground for the organisms, and lessen their irritative effect. Coliner[1] affirms that he has obtained good results in a series of cases by the use of orphol, that the stools became formed and regular, and that the pains disappeared. He states that the drug is a mixture of beta naphthol and oxide of bismuth, but in place of the burning taste of the former drug it is agreeable and slightly aromatic. Chaumier points out that it does not interfere with the stomach, but is broken up into its constituents in the intestines, where it acts as an astringent and antiseptic. Whether or no it has a direct action on the tubercle bacillus, it certainly appears to check secondary infection and fermentation. Thus, among other cases reported on, Coliner administered 15 grains every two hours after food to a phthisical patient, who suffered from diarrhœa, meteorism, and pain, in spite of the ordinary treatment. The symptoms were quickly relieved, and in three days constipation set in, the appetite improved, and the patient began to gain in weight. The bowels later on began to act once a day. The naphthol is chiefly excreted in the fæces, but a small amount passes off in the urine. The drug has of late been used to some extent for the diarrhœa of infants, for whom the pungent taste of beta naphthol by itself is great objection, since not only is there considerable pain if the latter substance is not swallowed at once in a liquid, but there is little certainty of the dose being really swallowed, and a difficulty is created in getting the child to take anything else from a spoon afterwards.

[1] The Charlotte Med. Journal, Feb., 1897; and the Allg. Med. Central Zeitung, 1896, No. 96.

